# Supplementary figures and images for: Combination of Ad-SGE-REIC and bevacizumab modulates glioma progression by suppressing tumor invasion and angiogenesis
Source: PLoS One. 2022 Aug 25;17(8):e0273242. doi: 10.1371/journal.pone.0273242 (PMC9409598; doi:10.1371/journal.pone.0273242)

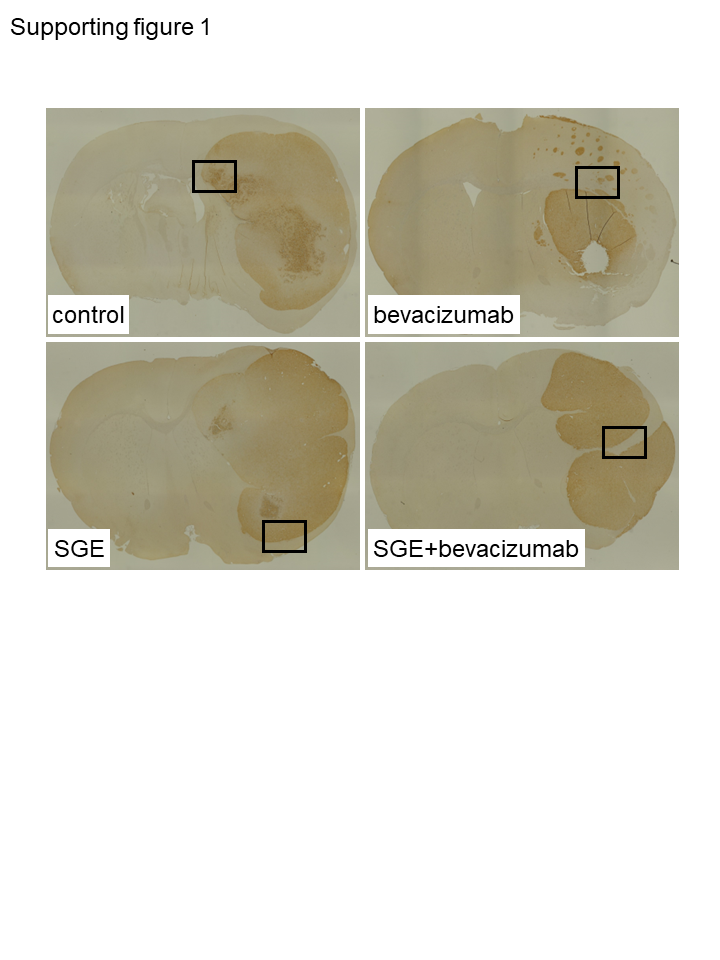

Supplement: S1 Fig — Marked area with black square is shown in Fig 5E. (TIF) [file pone.0273242.s001.tif]

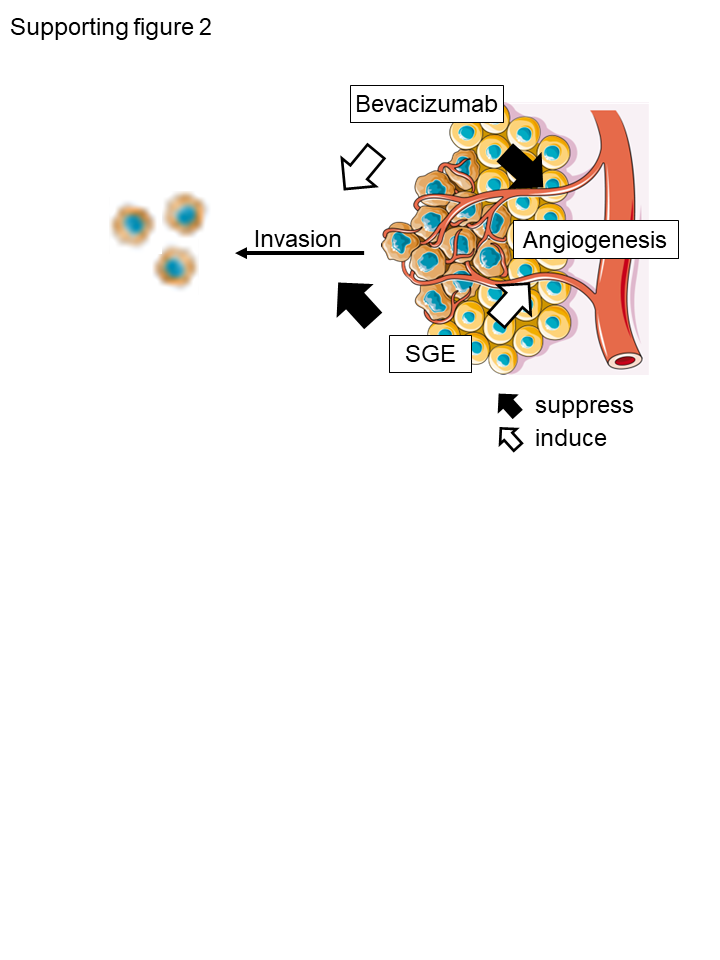

Supplement: S2 Fig — SGE reduces bevacizumab-induced invasion, and bevacizumab suppresses SGE-related angiogenesis. (TIF) [file pone.0273242.s002.tif]

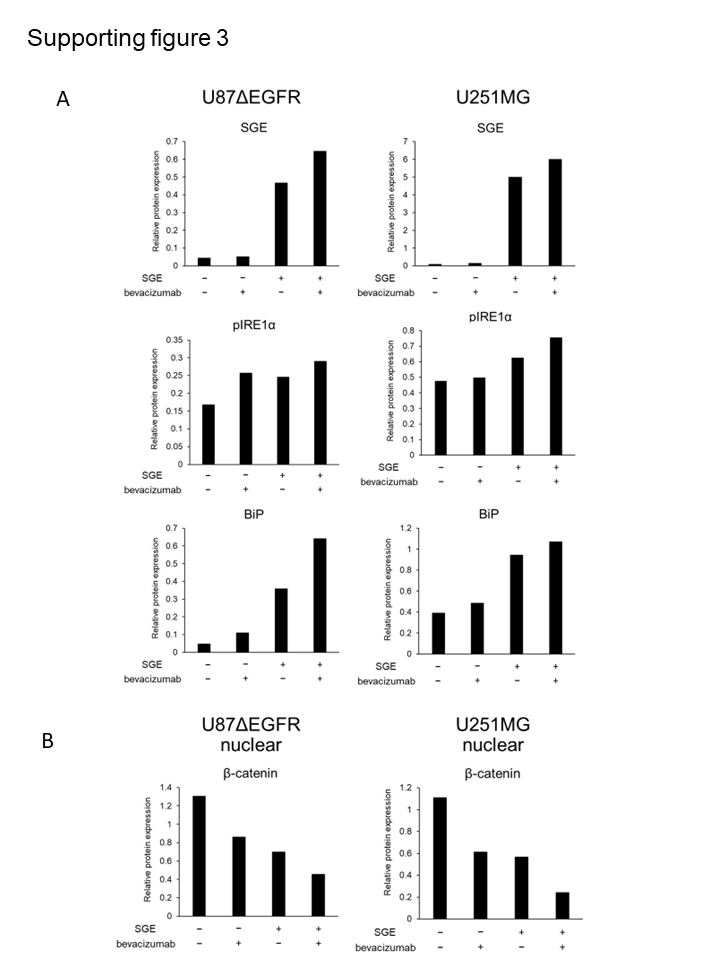

Supplement: S3 Fig — We evaluated the band densities of protein levels in each group using Image J (ver. 1.53r). (A) The relative protein expression of Bip and phosphorylated IRE1α were increased in the combination group compared with levels in the individual treatment groups. (B) Downregulation of β-catenin was observed in the combination therapy group compared with other treatment groups in each cell line. (TIF) [file pone.0273242.s003.tif]
