# Supplementary material for: Combination of Ad-SGE-REIC and bevacizumab modulates glioma progression by suppressing tumor invasion and angiogenesis
Source: PLoS One. 2022 Aug 25;17(8):e0273242. doi: 10.1371/journal.pone.0273242 (PMC9409598; doi:10.1371/journal.pone.0273242)

Figure 3

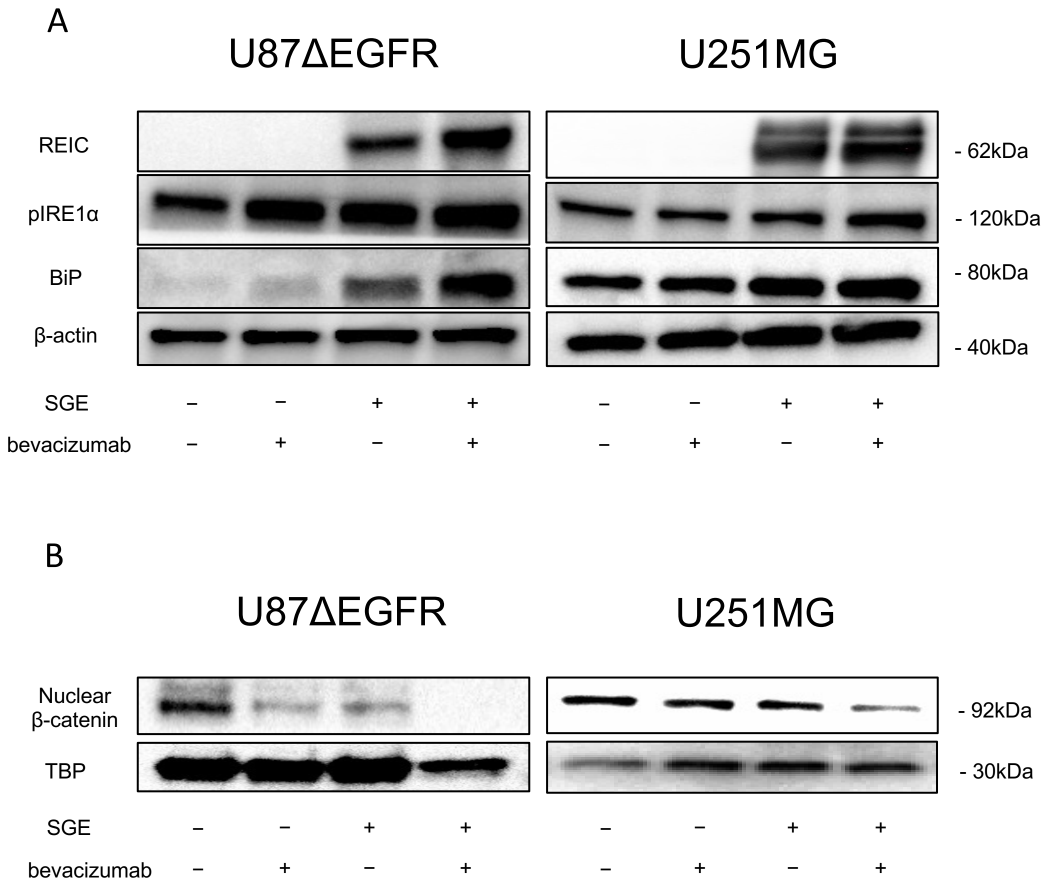
A
U87ΔEGFR
U251MG
REIC
pIRE1α
BiP
β-actin
- 62kDa
- 120kDa
- 80kDa
- 40kDa
SGE – – + + – – + +
bevacizumab – + – + – + – +
B
U87ΔEGFR
U251MG
Nuclear β-catenin
TBP
- 92kDa
- 30kDa
SGE – – + + – – + +
bevacizumab – + – + – + – +

U87ΔEGFR　REIC

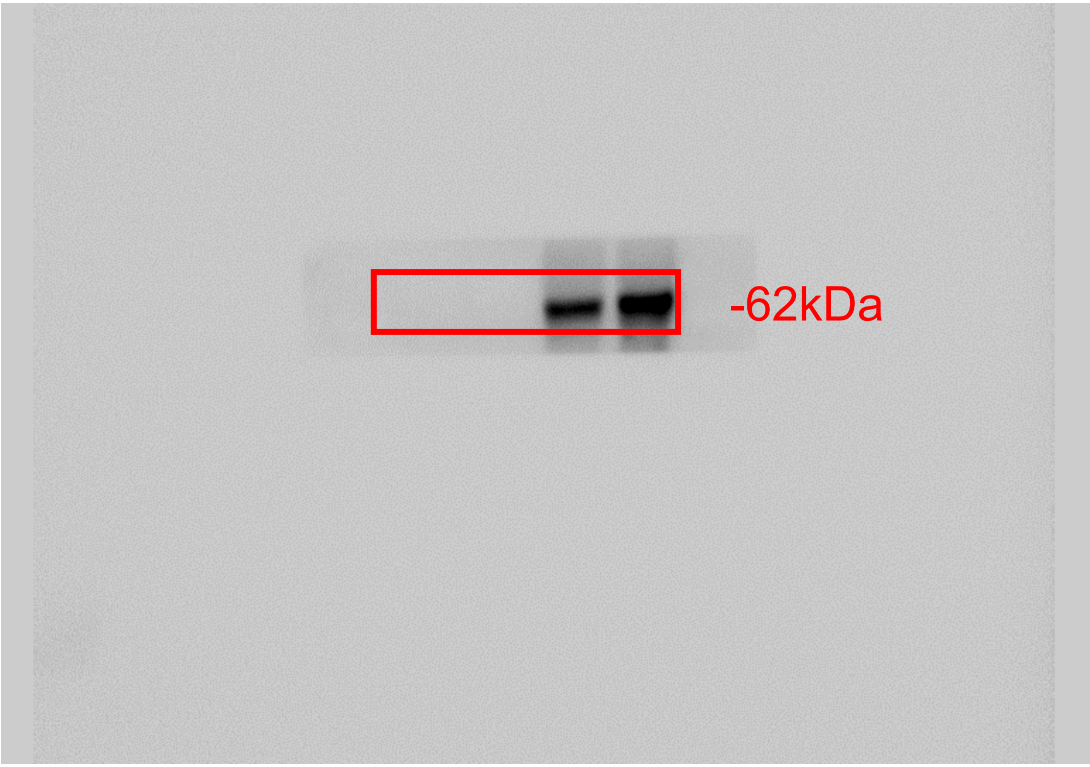

U87ΔEGFR pIRE1α

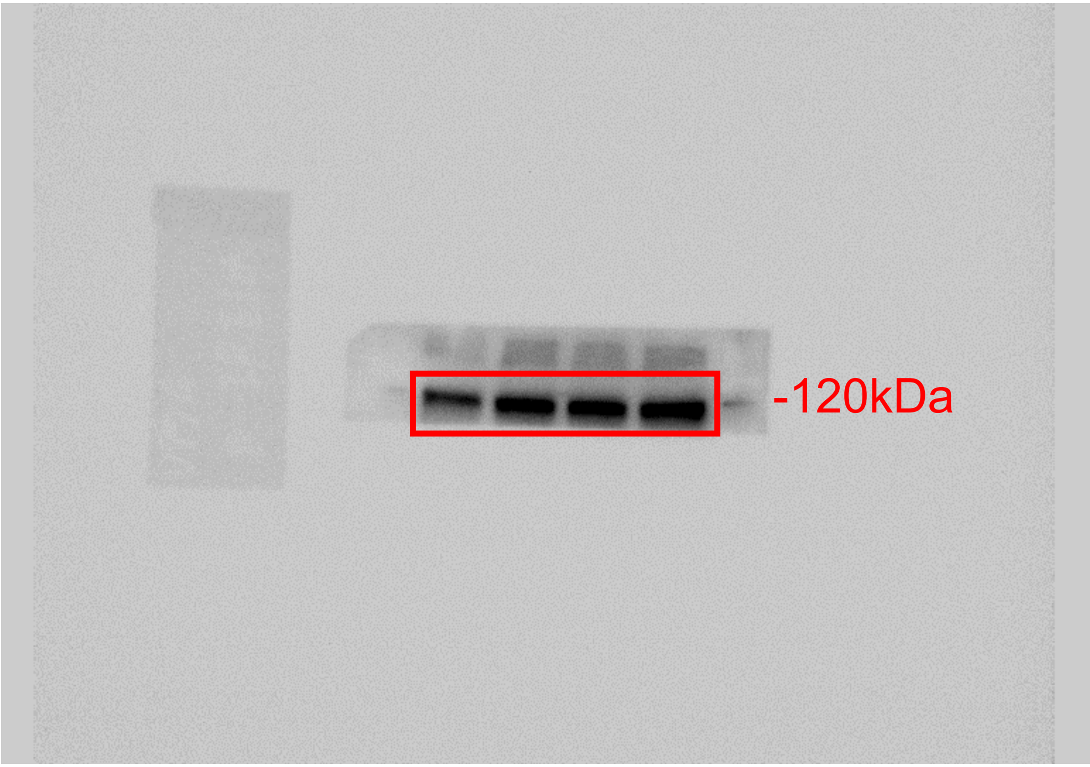

U87ΔEGFR   BiP

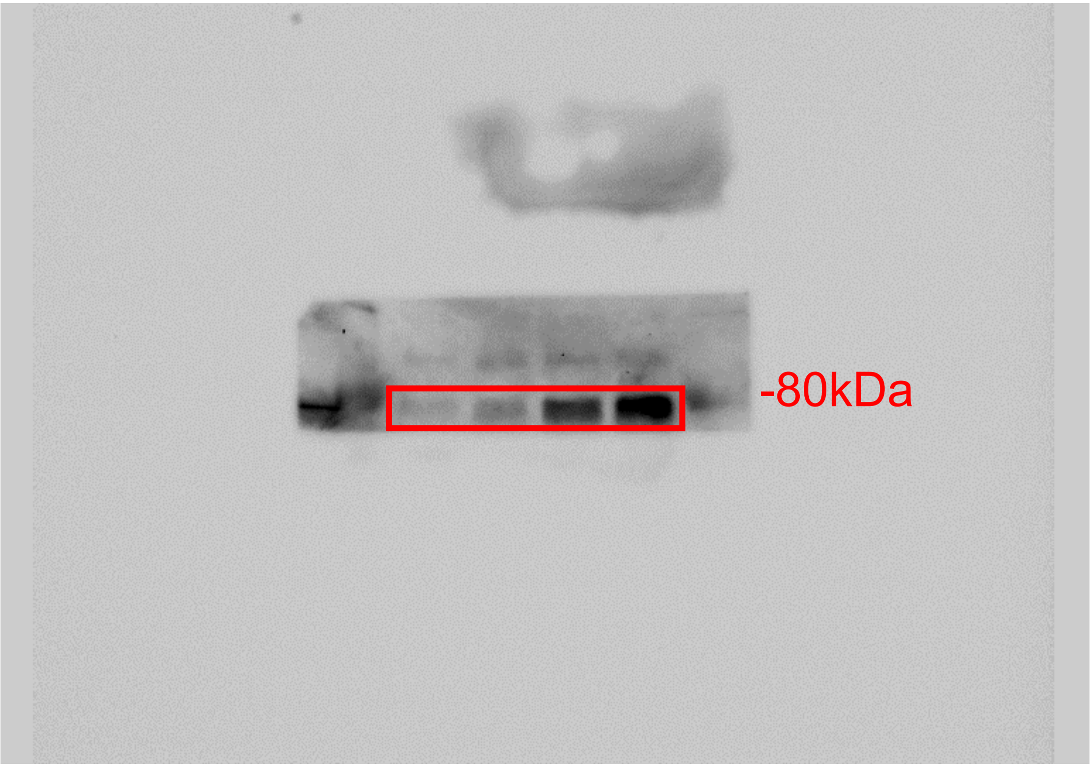

U87ΔEGFR β-Actin

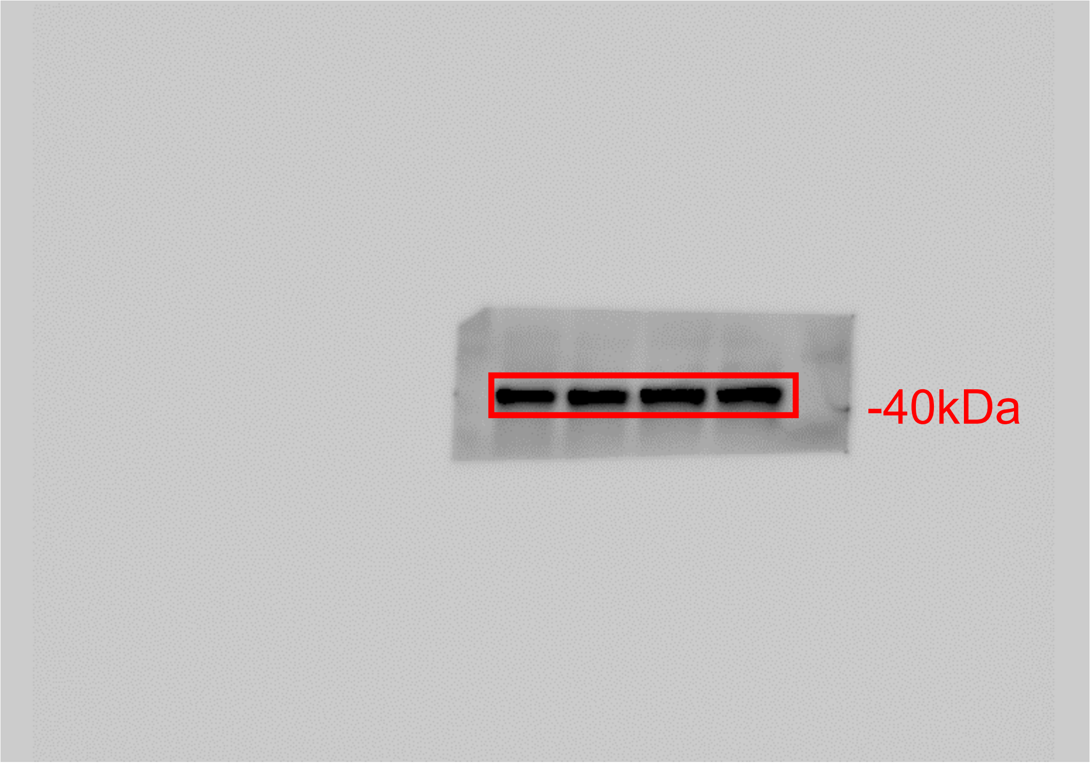

U251MG REIC

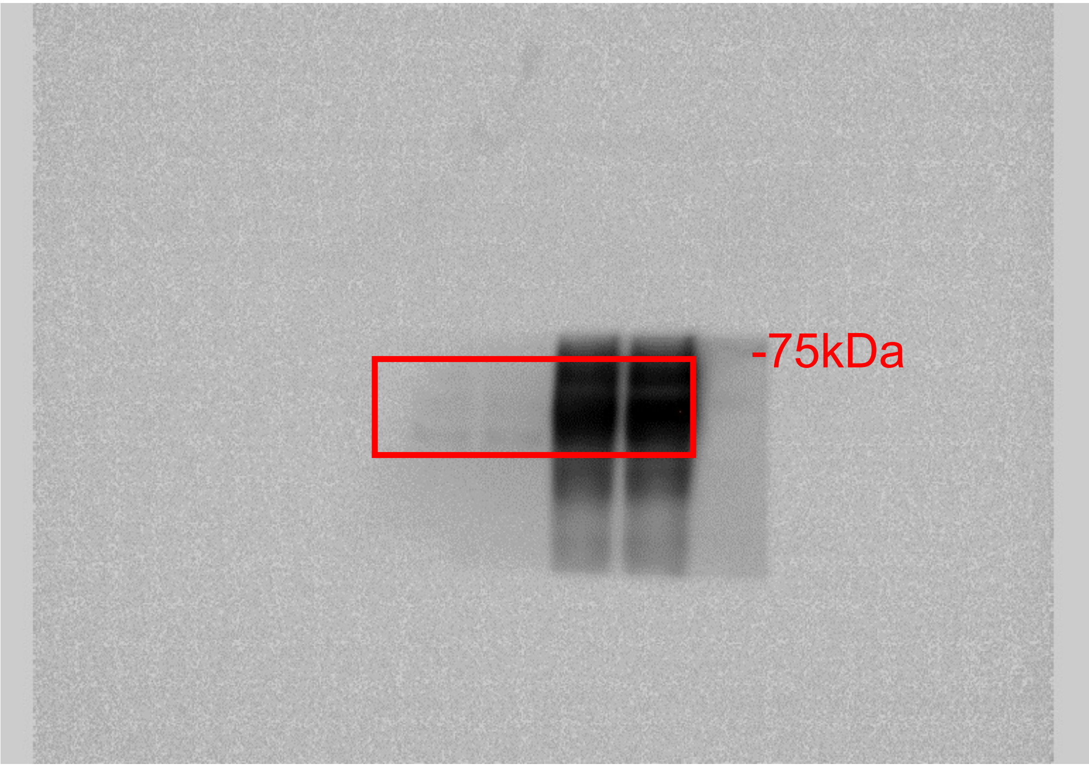

U251MG pIRE1α

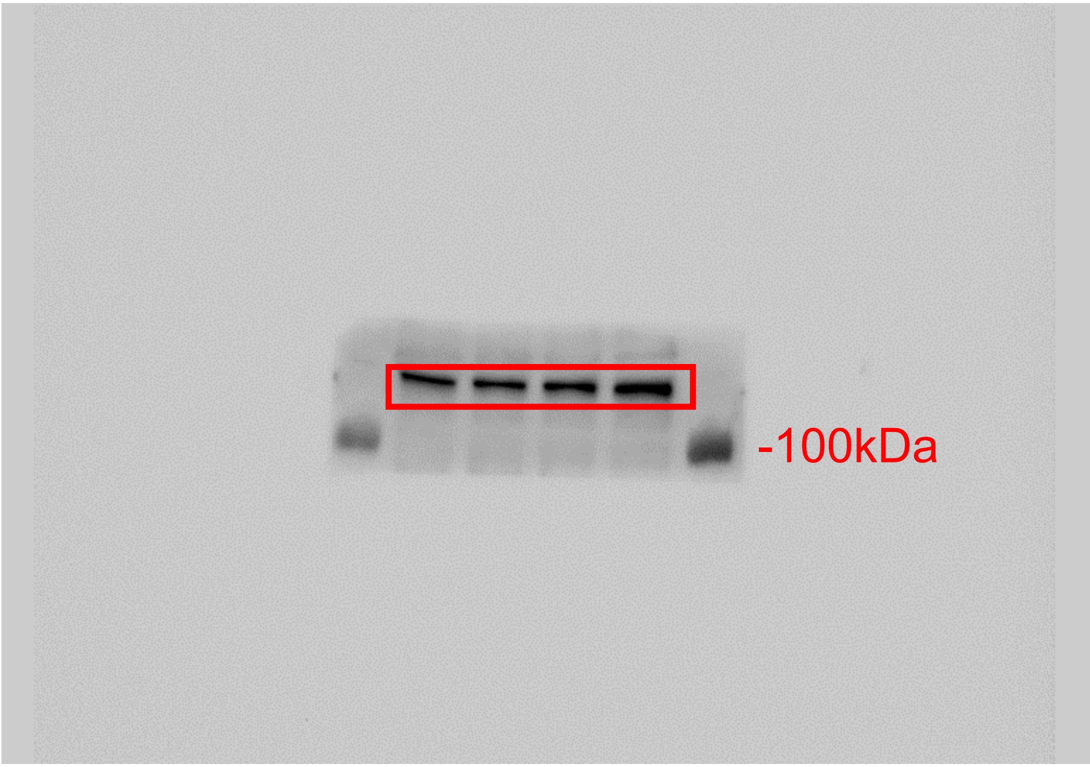

U251MG BiP

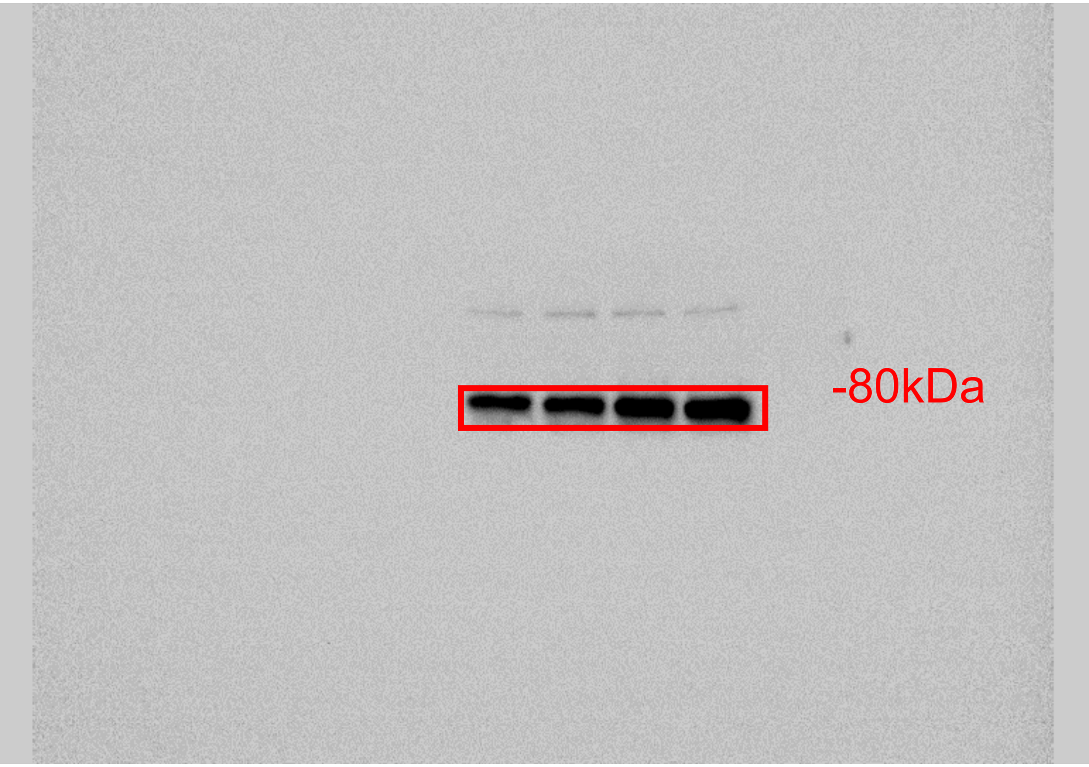

U251MG β-Actin

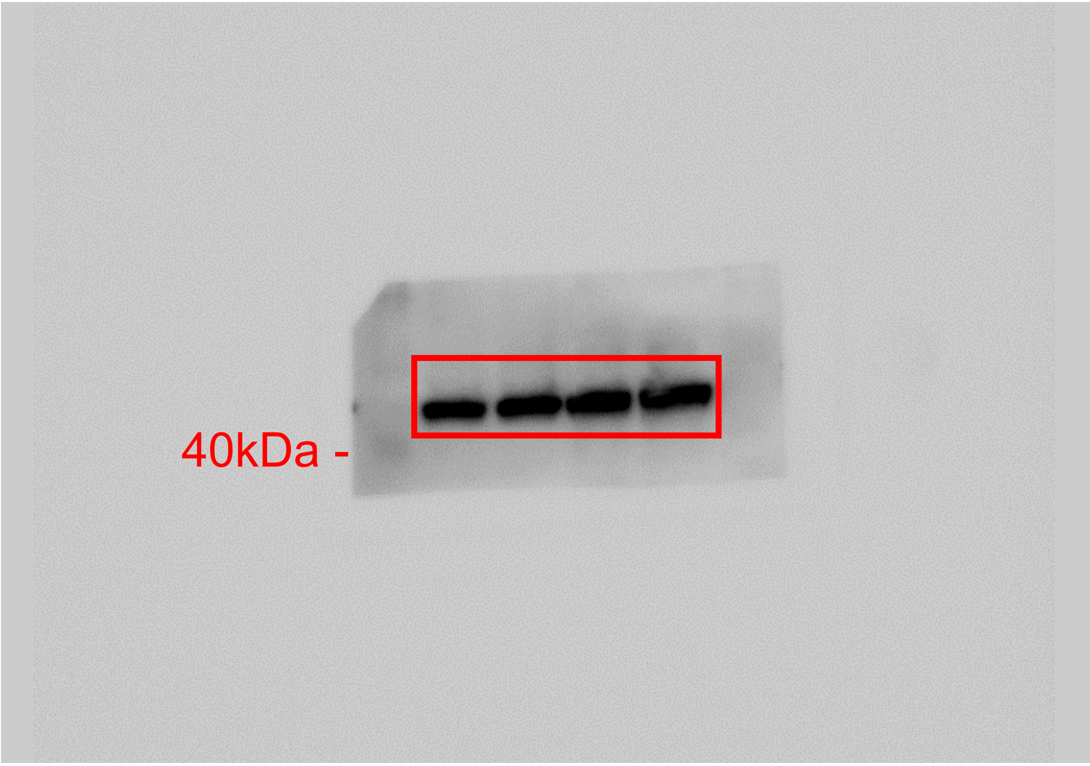

U87ΔEGFR nuclear β-catenin

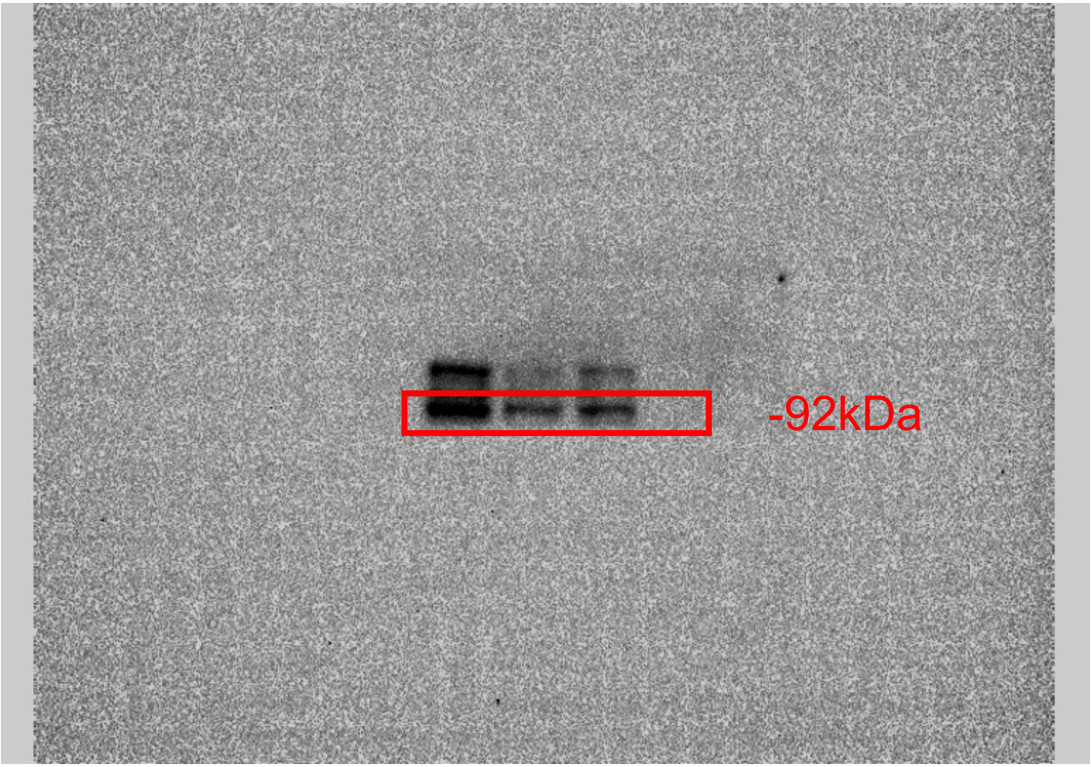

U87ΔEGFR nuclear TBP

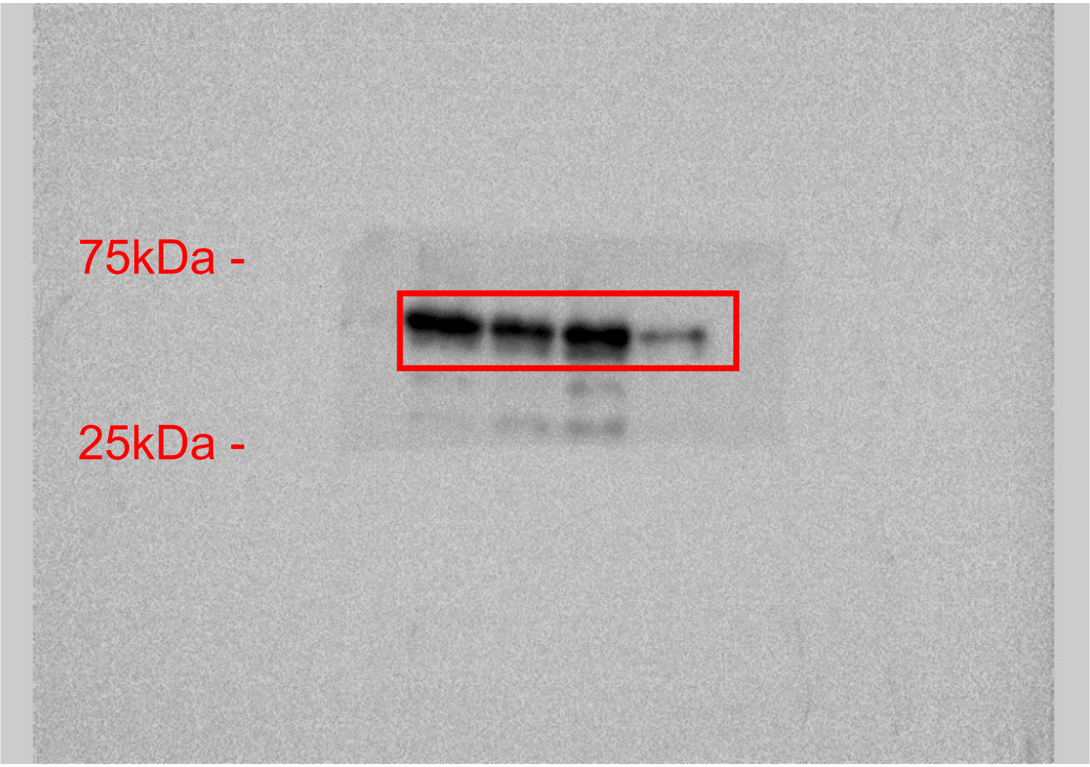

U251MG nuclear β-catenin

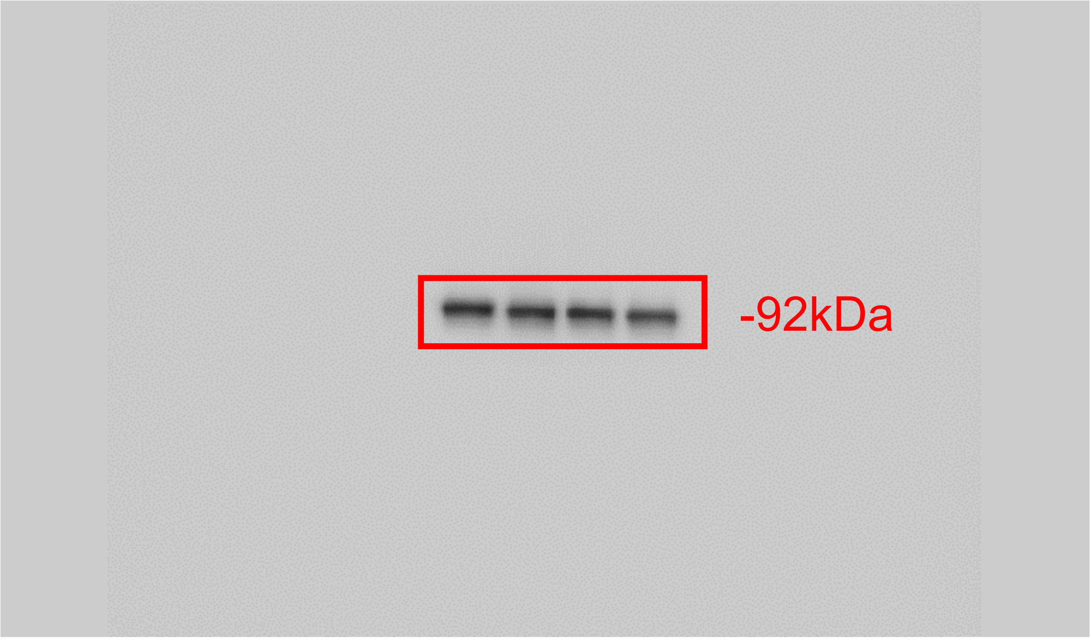

U251MG nuclear TBP

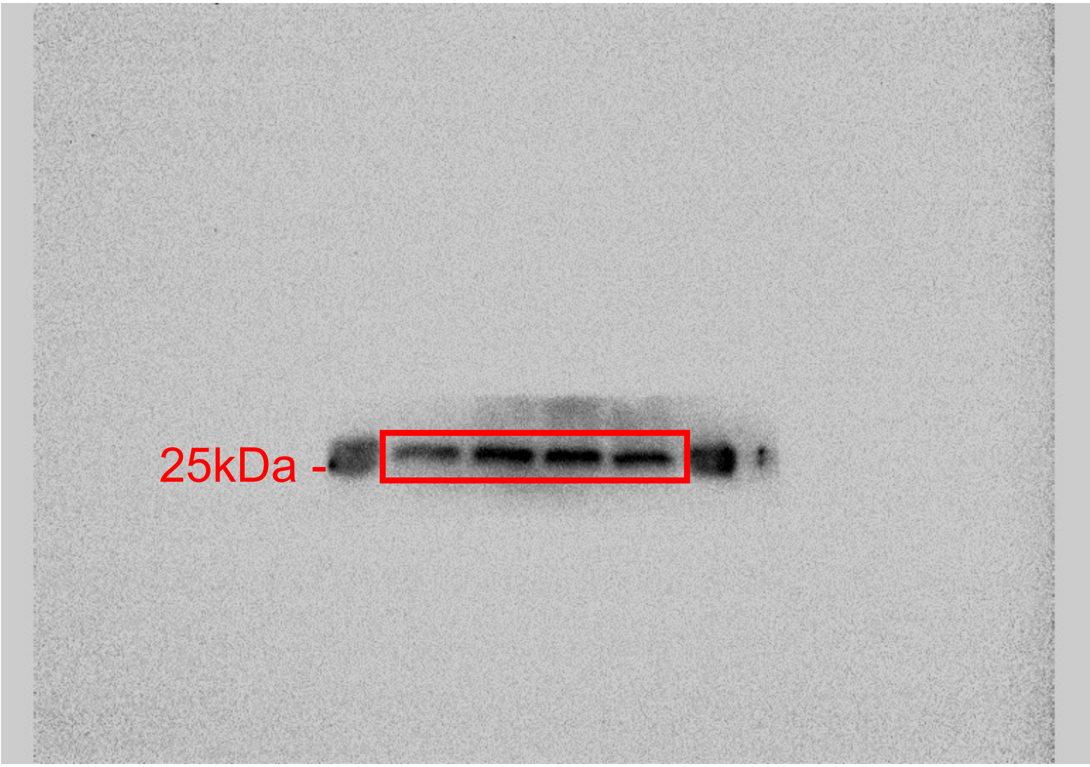

Supplement: S1 File — Boxes indicated parts used in the figure. (PDF) [file pone.0273242.s004.pdf]
