# Supplementary material for: Combination of Ad-SGE-REIC and bevacizumab modulates glioma progression by suppressing tumor invasion and angiogenesis
Source: PLoS One. 2022 Aug 25;17(8):e0273242. doi: 10.1371/journal.pone.0273242 (PMC9409598; doi:10.1371/journal.pone.0273242)
Supplement: Attachment — Submitted filename: Response to Reviewers 20220529.docx [file pone.0273242.s005.docx]

**Response to Reviewers**

We would like to express our appreciation to the reviewers for their insightful comments, which helped us significantly improve our paper.

**#1 The authors should better specify the mechanism of β-catenin reduction induced by the drug compound.**

Response:

Regarding the mechanism of β-catenin reduction, Behzadian reported that anti-VEGF therapy causes reduction of uPAR gene expression by transcriptional activation of β-catenin [1]. In addition, Moreau reported that β-catenin and NF-κB cooperate to regulate the uPA/uPAR system in cancer cells [2]. We performed preliminary western blot analysis and observed decreased uPAR protein expression in cells treated by the combination treatment (as shown below). Future studies are required to determine the precise mechanism.

References

1. M. Ali Behzadian, L. Jack Windsor, Nagla Ghaly, Ruth B. Caldwell_et_al. VEGF-induced paracellular permeability in cultured endothelial cells involves urokinase and its receptor. FASEB J. 2003 Apr;17(6):752-4. doi: 10.1096/fj.02-0484fje. Epub 2003 Feb 19. PMID: 12594181

2. Moreau M, Mourah S, Dosquet C. β-Catenin and NF-κB cooperate to regulate the

uPA/uPAR system in cancer cells. Int J Cancer. 2011 Mar 15;128(6):1280-92. doi:

10.1002/ijc.25455. PMID: 20473943.

**#2 In figure 1, the legends of panels C, D and E, F were inverted.**

Response:

We have corrected this information in the revised manuscript.

**#3 In figure 3A,** **the authors claimed significance between the combination group and individual treatments while those bands were not visually different. In figure 3B, the loading control TBP for U87DEGFR for the combination group was about half of the other groups, and thus it is unsupported to conclude that beta-catenin was down regulated.**

Response:

We appreciate the insightful observation. We quantified the band densities of protein expression of Bip and phosphorylated IRE1α between the combination group and individual treatments using Image J (ver. 1.53r). We also showed the downregulation of β-catenin in response to the combination therapy compared with other treatment groups in each cell line. We added these data in supporting figure 3.

**#4 Why was the advantage of using EGFR deleted U87 cells instead of regular U87?**

Response:

We would like to show the anti-glioma effects using glioma cell lines with high malignancy and U87ΔEGFR is one of the aggressive glioma cell lines. Moreover, TCGA database shows that glioblastoma patients have EGFR mutation or amplification, and therefore we considered that data in U87ΔEGFR cells may be more applicable to the clinic than data in U87 cells. We also reported the anti-glioma effects of Ad-SGE-REIC in U87ΔEGFR cells, and we would compare the higher effectiveness of combination therapy to Ad-SGE-REIC alone with the same cell line.

TCGA database

In addition

We added 2 authors to this study, because they helped our revise research.
